# Case Report: Fatal osimertinib-related interstitial lung disease during adjuvant therapy after left pneumonectomy in a patient with lung adenocarcinoma undergoing maintenance hemodialysis

**DOI:** 10.3389/fonc.2026.1860840

**Published:** 2026-07-27

**Authors:** Takahiro Kaki, Yuhei Harutani, Yoshimitsu Hirai, Hideto Iguchi, Issei Hirai, Mizuki Nishikawa, Daiki Kitahara, Yukiko Tsuchihashi, Kuninobu Kanai

**Affiliations:** 1 Department of Respiratory Medicine, Naga Municipal Hospital, Kinokawa, Wakayama, Japan; 2 Department of Thoracic and Cardiovascular Surgery, Wakayama Medical University, Wakayama, Wakayama, Japan; 3 Department of Breast and General Thoracic Surgery, Naga Municipal Hospital, Kinokawa, Wakayama, Japan; 4 Department of Human Pathology, Wakayama Medical University, Wakayama, Wakayama, Japan

**Keywords:** adjuvant therapy, EGFR-mutated non-small cell lung cancer, interstitial lung disease, maintenance hemodialysis, osimertinib, pneumonectomy

## Abstract

Osimertinib is recommended not only for advanced EGFR-mutated non-small cell lung cancer (NSCLC) but also as adjuvant therapy after complete resection, leading to increasing opportunities for its clinical use. However, interstitial lung disease (ILD) is a serious adverse event, and its incidence and severity have been reported to be higher in Japanese patients. We report a fatal case of osimertinib-related ILD that developed during adjuvant therapy in a patient undergoing maintenance hemodialysis after left pneumonectomy. A 71-year-old man was found to have a left upper lobe mass on chest computed tomography (CT) performed at the initiation of maintenance hemodialysis for nephrosclerosis. Transbronchial biopsy was nondiagnostic; however, lung cancer was strongly suspected clinically, and video-assisted thoracoscopic left pneumonectomy with mediastinal lymph node dissection was performed. Pathological examination revealed lung adenocarcinoma harboring an EGFR exon 19 deletion, pT2aN2bM0 (stage IIIB). Adjuvant osimertinib (80 mg/day) was initiated. Exertional dyspnea developed 37 days after treatment initiation, and chest CT on day 40 revealed peripheral ground-glass opacities in the remaining right lung. Osimertinib was discontinued, and empirical antibiotic therapy was initiated because both osimertinib-related ILD and infection were considered in the differential diagnosis. Shortly after admission, the patient developed rapidly progressive respiratory failure. Although steroid pulse therapy led to temporary improvement, the respiratory status deteriorated again, and the patient died on day 57 after osimertinib initiation despite an additional course of steroid pulse therapy. This case highlights that osimertinib-related ILD can become fatal not only in advanced or recurrent disease but also during curative-intent adjuvant therapy. Careful monitoring and prompt evaluation and intervention are particularly important in patients with poor respiratory reserve.

## 1 Introduction

Osimertinib is one of the standard treatment options for EGFR-mutated non-small cell lung cancer (NSCLC), not only in advanced or recurrent disease but also as adjuvant therapy after complete resection (1). In addition, its efficacy as consolidation therapy after definitive chemoradiotherapy for unresectable stage III disease has been demonstrated (2), leading to increasing opportunities for its clinical use.

Osimertinib-related interstitial lung disease (ILD) is an important adverse event, and its incidence and severity have been reported to be higher in Japanese patients than in other populations (3, 4). Herein, we report a case of grade 5 osimertinib-related ILD that developed during adjuvant therapy in a patient undergoing maintenance hemodialysis after left pneumonectomy.

## 2 Case presentation

A 71-year-old man was referred to our department in January 2024 after a left upper lobe mass was detected on chest computed tomography (CT) performed at the initiation of maintenance hemodialysis for end-stage renal disease secondary to nephrosclerosis. His medical history included chronic kidney disease requiring maintenance hemodialysis, cerebral infarction, and hypertension. He had smoked 20 cigarettes per day for only 1 year and had quit smoking at 21 years of age.

Before surgery, transbronchial lung biopsy using endobronchial ultrasonography with a guide sheath was performed twice from the left B3a. At the first bronchoscopy, neither histological examination nor cytology showed malignant findings. At the second bronchoscopy, histological diagnosis was again not established; however, cytology was classified as Class IV and reported as suspicious for non-small cell carcinoma. Therefore, NSCLC was suspected preoperatively.

Preoperative positron emission tomography (PET)-CT and contrast-enhanced thin-slice CT showed a primary tumor measuring approximately 38 mm, FDG uptake in hilar lymph nodes, and suspected central extension from the primary tumor toward the peribronchial region. In contrast, no apparent FDG uptake or significant enlargement was observed in mediastinal lymph nodes. Because the mediastinal lymph nodes were small, with a short-axis diameter of <5 mm, adequate sampling by endobronchial ultrasound-guided transbronchial needle aspiration (EBUS-TBNA) was expected to be technically difficult. Therefore, they were not considered clear targets for invasive pathological evaluation, and EBUS-TBNA was not performed. The preoperative clinical stage was assessed as cT2bN1M0 (stage IIB). Preoperative pulmonary function testing showed a forced expiratory volume in 1 s (FEV1) of 2.47 L (95.0% of predicted), a forced vital capacity (FVC) of 3.15 L (99.4% of predicted), and a diffusing capacity of the lung for carbon monoxide (DLCO) of 10.26 mL/min/mmHg (68.3% of predicted). Although a definitive histological diagnosis had not been obtained, NSCLC was strongly suspected based on the imaging and cytological findings; therefore, a surgery-first strategy was selected to establish a definitive diagnosis and provide curative treatment. Surgery was performed in April 2024.

No intraoperative frozen-section examination of either the primary tumor or mediastinal lymph nodes was performed. Because the disease had been assessed as cN1 preoperatively and there were no imaging findings suggestive of mediastinal lymph node metastasis, the surgical plan did not include discontinuing the operation if N2 disease was identified by intraoperative frozen-section examination.

Left upper lobectomy was initially planned. However, enlarged and infiltrative lymph nodes extending from the vicinity of the left main bronchial bifurcation to the segmental bronchi were identified intraoperatively. Because of these infiltrative lymph nodes, dissection distal to the left lower lobe bronchial bifurcation was difficult, and preservation of the lower lobe was judged impossible even with bronchoplastic reconstruction. Therefore, the procedure was converted to video-assisted thoracoscopic left pneumonectomy with mediastinal lymph node dissection (ND2a-2).

Pathological examination revealed lung adenocarcinoma harboring an EGFR exon 19 deletion, pT2aN2bM0 (stage IIIB), and R0 resection, including mediastinal lymph node dissection, was achieved. Adjuvant osimertinib was initiated at 80 mg/day. Exertional dyspnea developed 37 days after treatment initiation, and the patient made an unscheduled visit on day 40.

The clinical course is described using the day of osimertinib initiation as day 0. At presentation on day 40, the patient had an Eastern Cooperative Oncology Group performance status of 1. His body temperature was 37.6 °C, oxygen saturation was 96% on room air, and respiratory rate was 21 breaths/min. Fine crackles were heard on chest auscultation, but no cardiac murmur, peripheral edema, or physical findings suggestive of connective tissue disease were observed. No medications other than osimertinib had been changed within the preceding 6 months, and the patient had not used herbal medicines or supplements and had no apparent environmental exposure history.

The laboratory findings on admission are shown in **Table 1**. C-reactive protein (CRP) was elevated at 3.17 mg/dL, and lactate dehydrogenase (LDH) was elevated at 286 U/L. Krebs von den Lungen-6 (KL-6) and surfactant protein-D (SP-D) were elevated at 768 U/mL and 366 ng/mL, respectively. Blood and sputum cultures, urinary pneumococcal and Legionella antigens, and severe acute respiratory syndrome coronavirus 2 (SARS-CoV-2) polymerase chain reaction were all negative. No laboratory findings suggestive of vasculitis or connective tissue disease were identified.

**Table 1:** Laboratory findings on admission and arterial blood gas findings at respiratory deterioration.

Hematology			Blood chemistry			Immunological tests		
Parameter	Result	Unit	Parameter	Result	Unit	Parameter	Result	Unit
WBC	9170	/μL	CRP	3.17	mg/dL	ANA	<1:40	
Neut	67.2	%	LDH	286	IU/L	Anti-ARS antibody	<5.0	U/mL
Lymph	18.1	%	Cr	9.89	mg/dL	MPO-ANCA	<1.0	U/mL
Eos	3.4	%	BUN	54.3	mg/dL	PR3-ANCA	<1.0	U/mL
Hb	11.9	g/dL	KL-6	768	U/mL			
PLT	21.5 × 10^4^	/μL	SP-D	366	ng/mL			
			BNP	341	pg/mL			
Arterial blood gas*						Microbiological tests		
Parameter	Result	Unit				Test	Result	
pH	7.41					Sputum and blood cultures	Negative	
PaCO2	30.0	mmHg				Urinary pneumococcal antigen	Negative	
PaO2	59.0	mmHg				Urinary Legionella antigen	Negative	
HCO3^-^	18.7	mEq/L				SARS-CoV-2 PCR	Negative	
BE	-5.2	mEq/L						

*Arterial blood gas analysis was performed on day 42 under 4 L/min of oxygen via nasal cannula.

**Figure 1:**
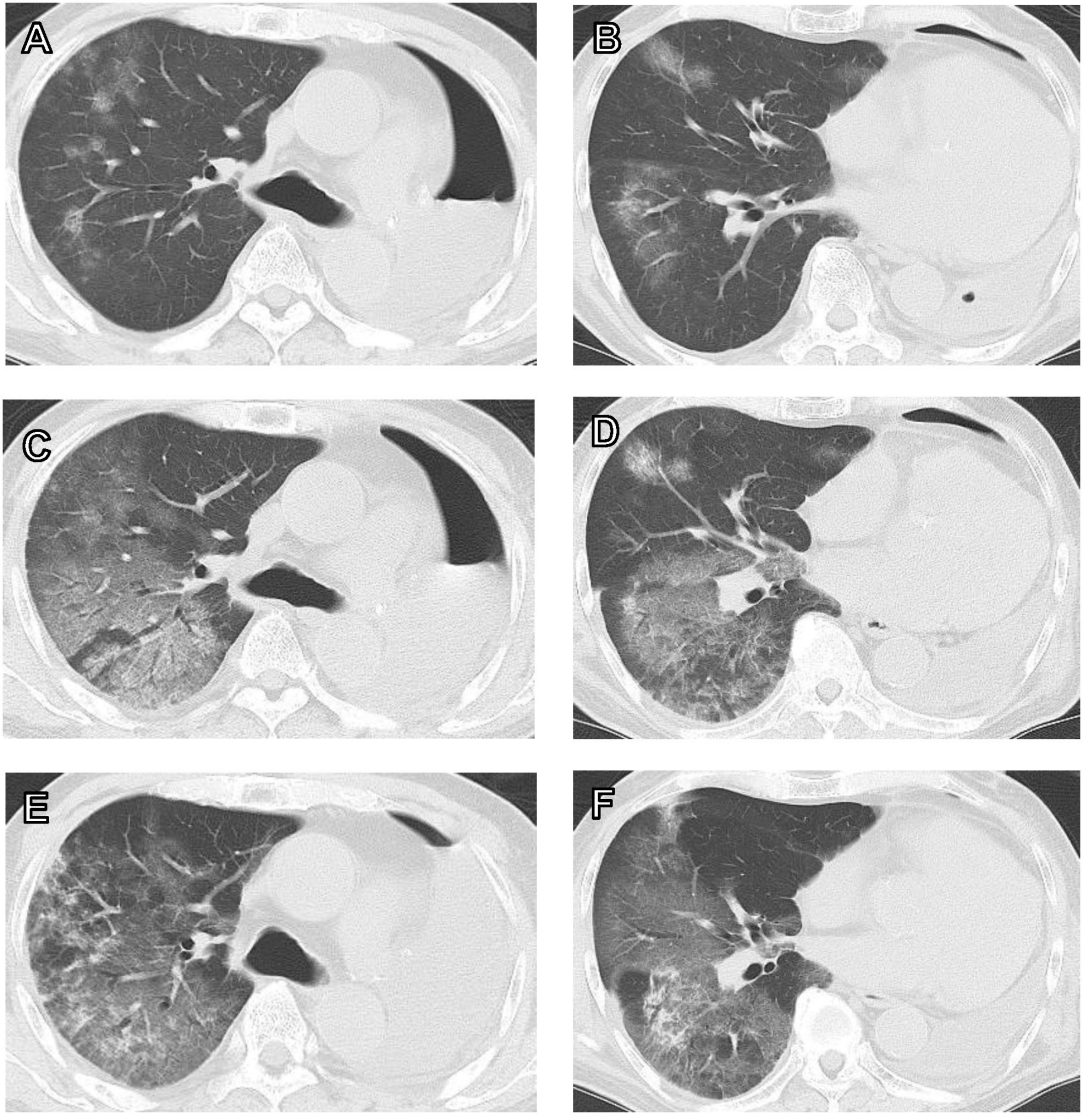
Chest CT findings during the clinical course. **(A, B)** Chest CT on day 40 at the unscheduled visit showing peripheral ground-glass opacities in the right lung, with patchy ground-glass opacities in the right upper and middle lobes and more extensive ground-glass opacities in the lower lobe. **(C, D)** Chest CT on day 42 showing rapid progression and more extensive ground-glass opacities in the right lung compared with day 40. **(E, F)** Chest CT on day 51 showing further progression of extensive ground-glass opacities in the right lung compared with day 42.

Preoperative chest CT showed no apparent interstitial abnormalities in the background lung. Chest CT at presentation revealed peripheral ground-glass opacities in the right lung, patchy in the right upper and middle lobes and more extensive in the lower lobe, suggesting an eosinophilic pneumonia/organizing pneumonia (EP/OP) pattern. Pleural effusion was present only on the pneumonectomy side, with no contralateral pleural effusion or apparent cardiomegaly (**Figure 1**).

Based on these findings, osimertinib-related ILD and pulmonary infection were considered in the differential diagnosis. Osimertinib was discontinued, and empirical antibiotic therapy with levofloxacin was initiated. Cardiogenic pulmonary edema was also considered but was judged unlikely based on the clinical course and imaging findings.

Despite antibiotic therapy, no improvement was observed, and oxygen supplementation was initiated on day 41. On day 42, respiratory failure progressed with worsening oxygenation and increased work of breathing, and high-flow nasal cannula therapy was initiated. Chest imaging showed rapid expansion of ground-glass opacities in the right lung, and clinical progression from the EP/OP pattern suggested on the initial CT to a diffuse alveolar damage (DAD)-like pattern was suspected. Bronchoscopy and bronchoalveolar lavage (BAL) were not performed because the risk of further respiratory deterioration associated with the procedure was judged to outweigh the potential benefit, given the rapid worsening of oxygenation and the presence of only one remaining lung after left pneumonectomy. Osimertinib-related ILD, clinically corresponding to grade 4 severity, was diagnosed, and methylprednisolone pulse therapy at 1 g/day was initiated for 3 days.

**Figure 2:**
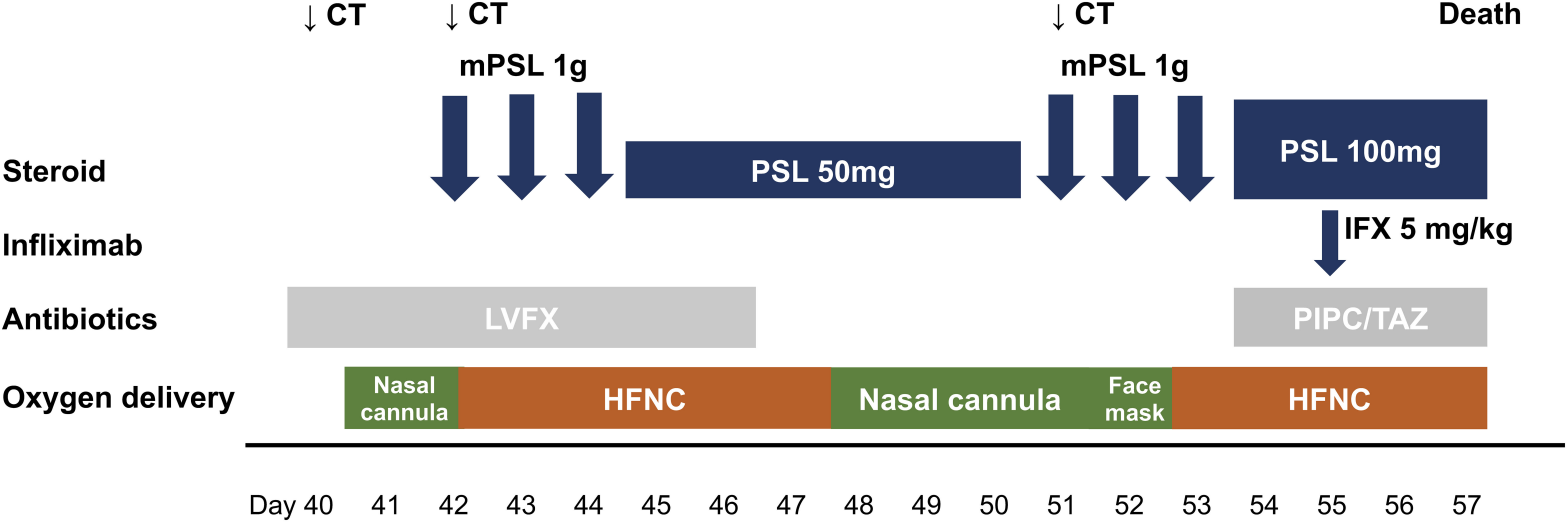
Clinical course. The day of osimertinib initiation was defined as day 0. The patient made an unscheduled visit on day 40, at which time osimertinib was discontinued and empirical antibiotic therapy was initiated. As respiratory failure progressed, high-flow nasal cannula therapy and methylprednisolone pulse therapy were initiated, followed by prednisolone treatment. Although the respiratory condition transiently improved, it subsequently worsened again. A second course of methylprednisolone pulse therapy and escalation of prednisolone were ineffective, and infliximab was administered on day 55. However, respiratory failure continued to progress, and the patient died on day 57. CT, computed tomography; mPSL, methylprednisolone; PSL, prednisolone; IFX, infliximab; LVFX, levofloxacin; PIPC/TAZ, piperacillin/tazobactam; HFNC, high-flow nasal cannula.

After the initial pulse therapy, the respiratory condition temporarily improved, and high-flow nasal cannula therapy was discontinued. Prednisolone was then continued at 50 mg/day (1 mg/kg/day). However, respiratory status worsened again on day 51. KL-6 and SP-D levels were 1404 U/mL and 820 ng/mL, respectively. Serum β-D-glucan was 27.5 pg/mL, and CMV C7-HRP was 1 cell; these findings did not actively support Pneumocystis jirovecii pneumonia or CMV pneumonia. A second course of methylprednisolone pulse therapy (1 g/day for 3 days) was initiated on the same day. No improvement in respiratory status was observed during the second pulse therapy, and high-flow nasal cannula therapy was required again on day 53. Prednisolone at 100 mg/day was administered from day 54 without improvement. Because the course was refractory, infliximab at 5 mg/kg was administered as additional immunosuppressive therapy on day 55. Nevertheless, respiratory failure continued to progress, and the patient died on day 57. The overall clinical course is shown in **Figure 2**.

To explore whether altered pharmacokinetics might have contributed to the lung injury, the plasma concentration of osimertinib was measured on day 56, 15 days after discontinuation and before hemodialysis. Measurement was performed using liquid chromatography-tandem mass spectrometry (LC-MS/MS) at the MST Division of Analytical Evaluation, Foundation for Promotion of Material Science and Technology of Japan (Tokyo, Japan). The plasma osimertinib concentration was 0.02 μg/mL

## 3 Discussion

Osimertinib-related ILD is an important adverse event. In clinical trials, the incidence of grade 5 ILD has been reported to be 0.4% overall and 0.5% in Japanese patients (2, 4–8). In a real-world study, the incidence of grade 5 ILD in Japanese patients was reported to be 1.1% (3). In the ADAURA trial, ILD occurred in 3% of patients, but all cases were grade 1 or 2, and no grade 3 or higher ILD was reported (5). Only a few real-world reports have described severe ILD developing during adjuvant osimertinib therapy (9, 10). Furthermore, to the best of our knowledge, reports of grade 5 ILD remain extremely limited, and we identified no reports explicitly describing a similar event in a patient after pneumonectomy. This case suggests that severe osimertinib-related ILD, which may be difficult to capture in clinical trials, can occur in real-world practice even in the adjuvant setting.

Chest CT and FDG-PET/CT are useful for evaluating mediastinal lymph node metastasis; however, negative imaging findings cannot completely exclude metastasis. The sensitivity and specificity of chest CT and FDG-PET/CT for diagnosing lymph node metastasis have been reported as 59% and 78%, and 77.4% and 90.1%, respectively (1). In this case, preoperative PET-CT and contrast-enhanced CT showed no apparent FDG uptake or significant enlargement of mediastinal lymph nodes. Nevertheless, given the cN1 status and primary tumor diameter of approximately 38 mm, invasive mediastinal staging, including preoperative EBUS-TBNA, could have been considered even for an imaging-negative mediastinum. Because the mediastinal lymph nodes were small, with a short-axis diameter of <5 mm, adequate sampling by EBUS-TBNA was expected to be technically difficult, and they were not considered clear targets for pathological evaluation at that time. The final diagnosis of pN2b illustrates the limitations of preoperative staging.

If N2 disease had been confirmed preoperatively, reconsideration of the treatment strategy, including surgical indication, neoadjuvant therapy, and definitive chemoradiotherapy, might have been necessary (1). However, given the EGFR exon 19 deletion and maintenance hemodialysis, it cannot be concluded that preoperative identification of N2 disease would have clearly established an alternative treatment strategy capable of avoiding pneumonectomy.

Although R0 resection was achieved, the tumor was classified as pT2aN2bM0 (stage IIIB) and was considered to carry a high risk of recurrence. Therefore, despite the markedly reduced respiratory reserve after left pneumonectomy, adjuvant osimertinib was considered a clinically reasonable option because the tumor harbored an EGFR exon 19 deletion. Standard treatment generally consists of cisplatin-based chemotherapy followed by adjuvant osimertinib. However, cisplatin-based chemotherapy was considered difficult in this patient because he was undergoing maintenance hemodialysis.

In this case, the timing after osimertinib initiation, imaging findings, elevations in KL-6 and SP-D, lack of response to antibiotic therapy, and feasible infectious disease evaluation all suggested that osimertinib-related ILD was the most likely clinical diagnosis. Because BAL and histological evaluation were not performed, infection could not be completely excluded. Although β-D-glucan and CMV C7-HRP at the time of relapse did not actively support Pneumocystis jirovecii pneumonia or CMV pneumonia, these tests had not been performed at the time of the initial deterioration. On comprehensive assessment using the World Health Organization-Uppsala Monitoring Centre causality assessment system, the causal relationship with osimertinib was judged probable/likely (11).

Smoking history has been reported as a risk factor for osimertinib-related ILD, and grade 5 cases have been reported more frequently in men and patients with poor performance status (3). Although this patient was male, few other reported risk factors were present, and his smoking exposure was minimal and remote. In addition, preoperative CT showed no apparent interstitial abnormalities in the background lung.

The fatal outcome in this case cannot be explained solely by osimertinib-related lung injury. Pneumonectomy is associated with reduced pulmonary function (12) and increased postoperative respiratory complications and mortality (13). In addition to perioperative respiratory complications, late readmission for respiratory failure after pneumonectomy has been reported (14). In the ADAURA trial, only a very small proportion of patients in the osimertinib group underwent pneumonectomy (1%) (5). Therefore, the safety of adjuvant osimertinib in patients after pneumonectomy remains insufficiently characterized. Physiological changes, including redistribution of ventilation and blood flow, may occur in the remaining lung after pneumonectomy, but whether these changes contribute to susceptibility to osimertinib-related ILD remains unclear. This case does not suggest that pneumonectomy increases the risk of developing osimertinib-related ILD. However, the markedly reduced respiratory reserve after left pneumonectomy may have contributed to the rapid progression of respiratory failure once osimertinib-related ILD developed.

The impact of maintenance hemodialysis on osimertinib safety cannot be determined from this case. Patients with end-stage renal disease undergoing maintenance hemodialysis are often excluded from clinical trials, and safety evidence in this population is limited. Previous reports have suggested that the pharmacokinetics of osimertinib during treatment in patients undergoing maintenance hemodialysis are generally similar to those in patients with normal renal function, with maximum plasma concentrations of approximately 563–800 nM after an 80-mg dose (15, 16). However, these reports describe concentrations measured during treatment.

In this case, the plasma concentration measured on day 56, 15 days after discontinuation and before hemodialysis, was 0.02 μg/mL (approximately 40 nM). Using the steady-state trough concentration of 389.1 nM and terminal half-life of approximately 48 h reported by Planchard et al., a simple first-order elimination calculation estimates a concentration of approximately 2 nM 15 days after discontinuation; thus, the observed concentration of approximately 40 nM appeared relatively high (17). However, no comparable data describing concentration changes during the 15 days after discontinuation following repeated dosing are available, and serial concentrations during treatment and after discontinuation were not obtained in this case. In addition, because changes in plasma concentrations before and after hemodialysis were not evaluated, the effects of hemodialysis on drug removal and exposure cannot be determined. Therefore, this single measurement alone cannot establish the effect of maintenance hemodialysis on drug exposure or whether high drug exposure contributed to ILD severity. Further investigation of osimertinib exposure and safety in patients undergoing hemodialysis is needed.

## 4 Conclusion

We report a fatal case of osimertinib-related ILD that developed during adjuvant therapy in a patient undergoing maintenance hemodialysis after left pneumonectomy. This case shows that severe osimertinib-related ILD can occur not only in advanced or recurrent disease but also during curative-intent adjuvant therapy. Careful monitoring and prompt evaluation and intervention are particularly important in patients with poor respiratory reserve.

## Patient perspective

Because of the patient’s fatal clinical course, the patient’s perspective could not be obtained.

## Data Availability

The original contributions presented in the study are included in the article/supplementary material. Further inquiries can be directed to the corresponding author.
